# Extended-Spectrum Beta-Lactamase- and Plasmidic AmpC-Producing *Enterobacterales* among the Faecal Samples in the Bulgarian Community

**DOI:** 10.3390/microorganisms12091777

**Published:** 2024-08-28

**Authors:** Petya Stankova, Lyudmila Boyanova, Daniela Atanasova, Sashka Mihaylova, Mariya Sredkova, Raina Gergova, Kalina Mihova, Rumyana Markovska

**Affiliations:** 1 Department of Medical Microbiology, Medical Faculty, Medical University, 1431 Sofia, Bulgaria; petya.stankova88@abv.bg (P.S.); l.boyanova@hotmail.com (L.B.); rtgergova@gmail.com (R.G.); 2 Medical Diagnostic Laboratory “Lina”, 8000 Burgas, Bulgaria; dany.atanasova@abv.bg (D.A.); sashkam@yahoo.com (S.M.); 3 Medical Centre “Exacta Medica”, Institute of Science and Research, Medical University, 5803 Pleven, Bulgaria; microvir@abv.bg; 4 Molecular Medicine Centre, Medical University, 1431 Sofia, Bulgaria; kalina_mihova@abv.bg

**Keywords:** faecal carriage, CTX-M, DHA, *Esherichia coli*, *Klebsiella pneumoniae*, Bulgaria

## Abstract

The aim of the present work was to genetically characterise cefotaxime-resistant enterobacteria isolated from community carriers in Bulgaria. In total, 717 faecal samples from children and adults in five medical centres in Sofia, Pleven and Burgas were examined. Antimicrobial susceptibility was evaluated by the disk diffusion method. *bla*_ESBL_ or plasmidic AmpC (pAmpC) genes were detected by PCR and sequencing. MLST and ERIC-PCR were used to detect clonal relatedness. Among the faecal samples, 140 cefotaxime-resistant enterobacteria were found. The most frequently detected species was *Escherichia coli* (77.9%, 109/140 samples), followed by *Klebsiella pneumoniae* (7.9%, 11/140). Among the isolates, *bla*_CTX-M-15_ (37.1%) was predominant, followed by *bla*_CTX-M-3_ (19.2%), *bla*_CTX-M-14_ (10%), and *bla*_CTX-M-27_ (4.3 %). Genes encoding pAmpC were observed in 11.4% (*bla*_DHA-1_, 16/140) and in 1.4% (*bla*_CMY-2_, 2/140). The frequency of ESBL and pAmpC producers among the subjects was 14.6% and 2.5%, respectively. No carbapenem-resistant isolates were found. Four main clonal complexes (CC131, CC10, CC38, and CC155) were detected among *E. coli* isolates. The most common type was ST131, phylogroup B2 (16.5%). The increased frequency of ESBL- and pAmpC-producing enterobacteria in the community is a prerequisite for treatment failures of the associated infections and a good background for further studies.

## 1. Introduction

*Klebsiella pneumoniae*, *Escherichia coli*, and *Enterobacter* spp. are among the most common difficult-to-treat Gram-negative bacteria due to the production of beta-lactamases, such as extended-spectrum beta-lactamases (ESBLs), chromosomal cephalosporinase class C (AmpC) enzymes, and carbapenemases, which can confer antibiotic resistance to cephalosporins and/or carbapenems [1,2,3]. These bacterial species cause a wide range of nosocomial infections such as bloodstream, pulmonary, urinary tract, and intra-abdominal infections [4]. *E. coli* and other enterobacteria such as *Klebsiella* spp. and *Enterobacter* spp. are part of the microbiota of the large intestines. 

ESBLs (class A from the Ambler scheme) first appeared in 1980 due to point mutations in genes encoding broad-spectrum SHV-1 and TEM-1 enzymes [5]. During the first decades, they were mostly associated with *K. pneumoniae* and *Enterobacter* spp. Since 2001, a new ESBL group (CTX-M enzymes) has appeared. They have increased stability to cefotaxime compared to the classic SHV and TEM variants that are mostly ceftazidime-resistant and have low minimal inhibitory concentrations (MICs) of cefotaxime. The CTX-M group, mainly *bla*_CTX-M-3_ and *bla*_CTX-M-15_, showed great transferability and faster spread all over the world. These genes are often located on specific plasmids such as IncL/M for *bla*_CTX-M-3_ and IncF for *bla*_CTX-M-15_ [5]. An association with insertion sequence (IS) elements such as IS26, IS*Ecp*1, and the increased mobilisation of CTX-M genes, enhancing their dissemination, has also been reported [5]. After their appearance, the prevalence of CTX-M producers rose not only in the hospitals, but also in the community, and mostly among *E. coli* isolates [5]. Usually, ESBLs are located on plasmids with genes for antibiotic resistance to other antibiotic groups such as aminoglycosides, quinolones, and many others. This allows for the selection of mutants resistant not only to beta-lactams but also to other antibiotic groups such as aminoglycosides and quinolones. In some cases, AmpC is mobilised on plasmids and transmitted with them. Several main families of plasmid-mediated AmpC (pAmpC), such as CMY, DHA, FOX, MOX, and ACC, have been identified [2]. The lack of appropriate treatment options for the infections caused by ESBL and AmpC producers has been followed by an increase in carbapenem use and emergence of carbapenemases, such as *Klebsiella-producing* carbapenemase (KPC), New Delhi Metalo beta-lactamase (NDM), Verona Integron Metallo-carbapenemase (VIM), and oxacillinases (OXA-48), with an increased level of antibiotic resistance in their producers [6]. 

The gastrointestinal tract of humans and animals is one of the main reservoirs of ESBL or carbapenemase producers, which can contribute to the spread of antibiotic resistance genes, both in hospital settings and in the community [7]. In a meta-analysis in 2021, the authors observed that the worldwide frequency of ESBL-producing intestinal *E. coli* isolates in the community was 16.5% [8]. The rapid increase in prevalence of ESBL and carbapenemase-producing microorganisms in the gastrointestinal tract is likely to be multifactorial due to inappropriate antibiotic use, prolonged hospital stays, and surgical interventions. Other possible sources are associated with animals (both meat consumption and pets), environmental factors, travels to developing countries, and direct transmission within households and society [9].

The aim of the present work was to investigate the frequency of ESBL-, carbapenemase-, and pAmpC-producing *Enterobacterales* in the community by collecting faecal samples from children entering kindergarten and adults on entry to employment, as well as to perform genetic characterisation of isolates.

So far, similar studies have been conducted in Bulgaria only on the faecal carriage of ESBL- and carbapenemase-producing *Enterobacterales* in the hospitals [10,11] but not in the community.

## 2. Materials and Methods

### 2.1. Bacterial Isolates

**Figure 1:**
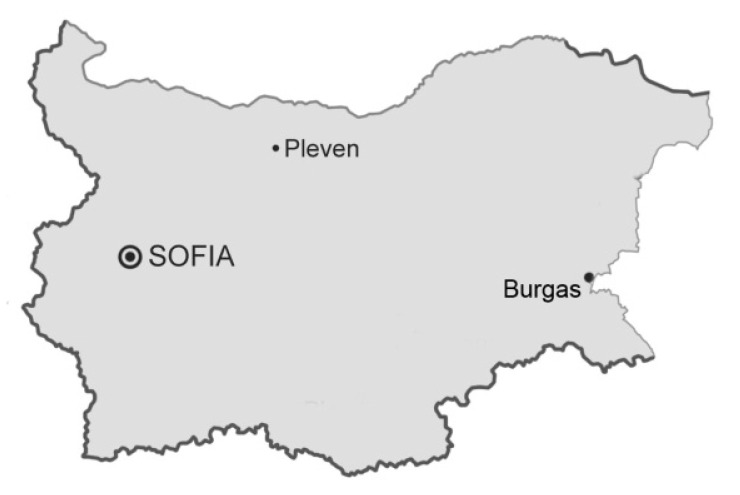
Area or location of the towns included in this study.

This study was conducted among outpatients in five medical centres, including one centre in Pleven, one in Burgas, and three in Sofia (Figure 1). The faecal samples of 717 subjects from the community were studied in the period January 2018–March 2019. The faecal samples were obtained during routine diagnostic work of the centres (microbiology laboratories). The subjects included children who were screened for admission to kindergartens and adults who applied for jobs in kindergartens or food preparation institutions. The adults and the parents of the children signed a written informed consent. The faecal samples were obtained in sterile containers and transferred to the microbiology laboratory within two hours and were inoculated immediately on MacConkey agar (Oxoid Ltd., Basingstoke, Hants, UK) with 1 mg/L cefotaxime (ICN Biomedicals Inc., Aurora, OH, USA). After overnight cultivation, the plates were examined for bacterial growth. One separated bacterial colony was subcultured. Only one isolate per patient was examined. The colonies were screened by Gram staining and oxidase tests before identification. Every colony type (lactose-positive or lactose-negative), showing Gram-negative rods in the Gram-stained smear and an oxidase-negative result, was further identified. Bacterial isolates were identified by routine biochemical tests (indole test, citrate utilisation test, urease, lysine and ornithine decarboxylase, and growth in Kligler medium (Bulbio, NCIPD, Sofia, Bulgaria)) and then were confirmed by matrix-assisted laser desorption ionisation time-of-flight mass spectrometry (MALDI-TOF MS) (VITEK MS (bioMérieux, Marcy L’Étoile, France)). This is a rapid and accurate method for identification of microorganisms on the basis of proteomic fingerprinting using high-throughput MALDI-TOF mass spectrometry [12]. 

### 2.2. Phenotypic Methods for ESBL/Carbapenemase Detection: Antimicrobial Susceptibility Testing

Putative ESBL production was demonstrated by the double-disk synergy method [13]. Briefly, a disk of amoxicillin-clavulanate (20/10 μg) was placed in the centre of an inoculated plate with Müller–Hinton II agar (Liofilchem, Roseto d. Abruzzi (TE), Italy), and around it, disks of third-generation cephalosporins (cefotaxime, and ceftazidime, Oxoid Ltd., Basingstoke, Hants, UK) were placed at 20 mm centre-to-centre on the plate. The increase in the inhibitory zone of the third-generation cephalosporins (synergism between third-generation cephalosporins and amoxicillin-clavulanate) was considered as positive for ESBL production. 

Antimicrobial susceptibility testing was performed by the disk diffusion method on Müller–Hinton II agar (Liofilchem, Roseto d. Abruzzi (TE), Italy) according to the guidelines of the European Committee on Antimicrobial Susceptibility Testing (EUCAST), 2020 [14]. The interpretation of the results was according to the EUCAST (2020) guidelines [15]. The following antibiotics were tested: beta-lactams including amoxicillin/clavulanic acid 30 µg (AMC), ceftazidime 10 µg (CAZ), cefotaxime 5 µg (CTX), cefepime 30 µg (FEP), cefoxitin 30 µg (FOX), imipenem 10 µg (IMP), meropenem 10 µg (MEM), piperacillin/tazobactam 36 µg (PIP/TAZ), aminoglycosides including tobramycin 10 µg (TOB), gentamicin 10 µg (GEN), amikacin 30 µg (AMK), trimethoprim/sulfamethoxazole 25 µg (SXT), quinolones including ciprofloxacin 5 µg (CIP), levofloxacin 5 µg (LVX), as well as chloramphenicol 30 µg (CHL) (Oxoid Ltd., Basingstoke, Hants, UK).

### 2.3. Molecular–Genetic Methods for Beta-Lactamase Identification

Presumptive ESBL producers (based on the double-disk synergy method) were confirmed by PCR with SHV and CTX-M group-specific primers [16]. All isolates showing antagonism or lack of synergism by the double-disk synergy method, and all cefoxitin-resistant *E. coli* and *K. pneumoniae* isolates, as well as those with innate AmpC production (all *Enterobacter* spp., *Morganella morganii*, *Citrobacter freundii* complex, and *Hafnia alvei*, *n* = 35) were tested for the presence of genes encoding plasmidic AmpC enzymes such as *bla*_DHA_, *bla*_CMY_, *bla*_MOX_, *bla*_FOX_, and *bla*_ACC_ as previously described [17]. PCR primers (Metabion, Planegg, Germany) and annealing temperature for the reactions are shown in Table S1. Primers and annealing temperature for the sequencing reactions (*bla*_CTX-M-1-group_, *bla*_CTX-M-9_ group [11,16], *bla*_CMY_ and *bla*_DNA_ [11,18]) are shown in Table S2. Prime Taq polymerase (GenetBio Inc., Daejeon, Republic of Korea) and reaction buffer with 2 mmol MgCl (GenetBio Inc., Daejeon, Republic of Korea) were used. Nucleotide sequences were analysed using Chromas Lite 2.01 (Technelysium Pty Ltd., Brisbane, Australia) and DNAMAN software, version 8.0 (Lynnon BioSoft, Vaudreuil-Dorion, GM, Canada).

### 2.4. Molecular Typing

Clonal relatedness was detected via the MLST and ERIC-PCR methods. For ERIC PCR, ERIC 1R, and ERIC 2 primers were used [19] (Table S1), and the protocol was as described previously [16]. The isolates with a difference of up to 1 band in the ERIC profiles were considered to be clones. 

The Pasteur scheme was used for *K. pneumoniae* MLST typing, and the Achtman scheme was applied for *E. coli.* For *E. coli* isolates, the assignment to allelic numbers and sequence types (STs) was performed according to the MLST database [20]. The primers and protocols used were as described by Wirth et al. [21]. The primers are shown in Table S3.1.

For the *K. pneumoniae* isolates, primers, protocols and assignment to allelic numbers and sequence types (STs) were carried out as described in the MLST database [22] and by Diancourt et al. [23]. The primers are shown in Table S3.2. A clonal complex was defined as a group of two or more independent isolates that share six identical alleles. The MLST dataset was generated using PhYLOViZ Online [24].

Detection of the specific hypervirulent O25b:H4-ST131 clone was performed with allele-specific PCR for the *pabB* gene as previously described [25]. The representative isolates according to the species, ERIC types, and the genes detected were subjected to MLST. They included 10 of the 12 *K. pneumoniae* and 53 of the 109 *E. coli* isolates.

The phylotyping of *E. coli* isolates was performed as described previously [26].

### 2.5. Statistical Analysis

Differences in the frequency of ESBL or pAmpC between the children and adults were assessed by the chi-square test/Fisher’s exact test GraphPad [27]. The results were considered significant when the *p* value was <0.05.

## 3. Results

### 3.1. Bacterial Isolates

Among the faecal samples of 717 community carriers, 140 isolates resistant to third-generation cephalosporins (19.5%, 140/717) were detected on the selective media. The isolates identified in the 408 children and 309 adults were 93 and 47 (22.8% and 15.2%), respectively. The isolates were *E. coli* (*n* = 109), *K. pneumoniae* (*n* = 11), *Enterobacter cloacae* complex (*n* = 8), *C. freundii* complex (*n* = 6), *M. morganii* (*n* = 2), and *H. alvei* (*n* = 4). (Table 1).

The predominant isolates in the present study were *E. coli* (77.9%, 109/140 isolates), followed by *K. pneumoniae* (7.9%, 11/140).

### 3.2. Phenotypic Methods for ESBL/Carbapenemase Detection: Antimicrobial Susceptibility Testing

The presumptive ESBL production was detected by the double disk synergy test on 105 isolates (14.6%, 105/717, of all subjects) and included *E. coli* (92 isolates), *K. pneumoniae* (10), *C freundii* complex (2), and *E. cloacae* complex (1). Thirty-five isolates were negative.

**Figure 2:**
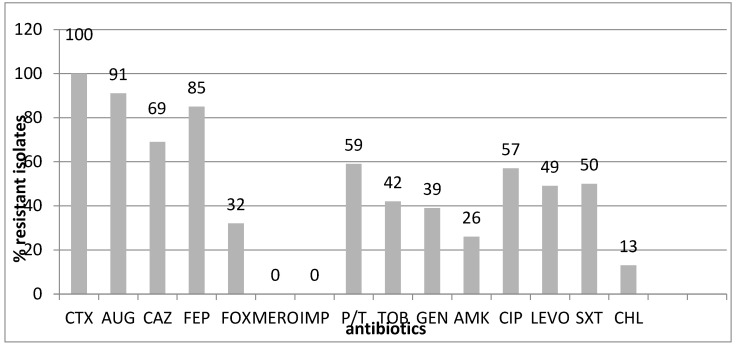
The percentage of antibiotic-resistant and intermediate enterobacteria (*n* = 140) in this study. Abbreviations: cefotaxime (CTX), amoxicillin/clavulanic acid (AUG), ceftazidime (CAZ), cefepime (FEP), cefoxitin (FOX), meropenem (MERO), imipenem (IMP), piperacillin/tazobactam (P/T, tobramycin (TOB), gentamicin (GEN), amikacin (AMI), ciprofloxacin (CIP), levofloxacin (LEVO), co-trimoxazole (SXT), chloramphenicol (CHL).

The antibiotic resistance of the isolates (*n* = 140) is shown in Figure 2.

Our isolates exhibited the following antibiotic resistance rates to non-beta-lactam antibiotics: 26–42% for aminoglycosides and 49–57% for fluoroquinolones (Figure 1). Trimethoprim/sulfamethoxazole resistance was also high (50%). No carbapenem-resistant isolates were found in this study.

### 3.3. Molecular–Genetic Methods for Beta-Lactamase Identification

PCR and sequencing confirmed the presence of *bla*_ESBL_ in 14.6% (105/717) of the isolates. *bla*_CTX-M_ prevailed with *bla*_CTX-M-15_ in 37.1% (52/140) of the cefotaxime-resistant isolates (Table 2). The frequency of *bla*_CTX-M-3_ was 19.3% (27/140 isolates), followed by *bla*_CTX-M-14_ in 10% (14/140), *bla*_CTX-M-27_ in 4.3% (6/140), *bla*_CTX-M-1_ in 2.1% (3/140), and *bla*_CTX-M-9_ in 0.7% (1/140). Only two isolates had *bla*_SHV-12_ 1.4% (2/140) (Table 2).

Eighteen isolates (2.5%, 18 of 717 patients investigated) were positive for genes encoding plasmidic AmpC. We detected the presence of *bla*_DHA-1_ in 11.4% of them (16/140, including 15 *E. coli* and one *K. pneumoniae* isolate). Two *E. coli* isolates (1.4%, 2/140 isolates) were positive for *bla*_CMY-2_.

Seventeen isolates (seven *E. cloacae*, four *C. freundii*, and all *M. morganii* and *H. alvei* isolates) were negative for ESBL genes and resistant to cefoxitin, and we accepted them as presumptive innate AmpC producers. They were found in 2.4% (17/717 patients) and included *E. cloacae* complex (7 isolates), *H. alvei* (4), *C. freundii* complex (4), and *M. morganii* (2).

The frequency of ESBL-producing *Enterobacterales* among the community carriers was 14.6 % (105/717 isolates). Among the adults, the percentage was 11.7% (36/309 isolates), and that among the children was 16.9% (69/408). The difference did not reach statistical significance (*p* = 0.09). The frequency of pAmpC was 2.5% (18/717 isolates). The pAmpC frequency in the adults and children was 1.9% (6/309) and 2.9% (12/408), respectively. The difference for pAmpC was not statistically significant either (*p* = 0.47).

### 3.4. Molecular Typing

The isolates positive for ESBL or plasmid AmpC genes were typed by ERIC-PCR. For each isolate, recognizable ERIC profiles of 7 to 14 bands were generated.

**Figure 3:**
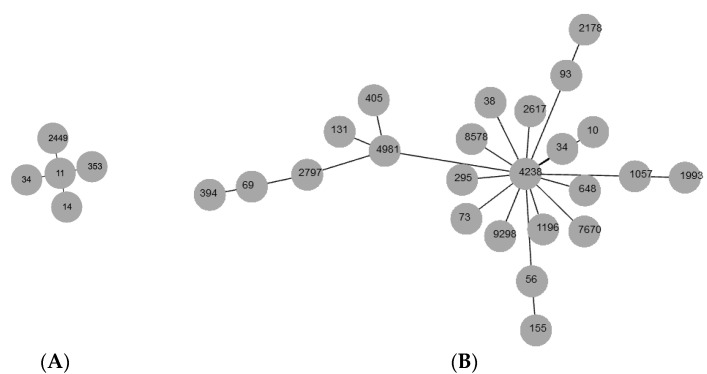
The MLST dataset was generated by PHYLOViZ and indicates the observed sequence types (STs) among *E. coli* and *K*. *pneumoniae* isolates. The numbers in the circles correspond to the STs. (**A**) *K. pneumoniae*, (**B**) *E. coli*.

In *K. pneumoniae* isolates (*n* = 11), 10 ERIC types were identified. From each ERIC type, the MLST types were determined. Four of the isolates were not typed. Table 3 shows the ST types of *K. pneumoniae* isolates and the corresponding ESBL or pAmpC genes detected. The MLST dataset of ST types was created (Figure 3A). ST14 and ST11 exhibited only one allele difference.

A total of 56 ERIC types were identified among 109 *E. coli* isolates; 43 of them showed unique profiles, and the others made clusters with 2 to 18 members. MLST types and the corresponding enzymes in *E. coli* are shown in Table 4. In the present study, we observed four main Clonal Complexes (CC): CC131, CC10, CC38, and CC155. The most common ST type was 131 of phylogroup B2 (16.5%, 18/109), followed by CC10 (ST4981, ST4238, ST10, and ST34) in phylogroup A (15.6%, 17/109), CC38 (ST38) (10%, 11/109) of group D, and ST155 and ST51 (CC155) of group D (4.6%, 5/109). The MLST dataset was created (Figure 3B). The positive *pabB* allele-specific PCR confirmed that all 18 isolates from the ST131 clone were members of the O25b:H4 clade.

The phylotypes and their association with ST types are shown in Table 5. Most of the isolates belonged to the group D 34.9% (38/109 isolates), and the frequencies of phylogroups A, B2, and B1 were 29.4% (32/109), 25.7% (28/109), and 10.1% (11/109), respectively.

## 4. Discussion

The digestive tract is the main reservoir of resistant enterobacteria and a hot spot for the exchange of genes for antibiotic resistance. For the first time, intestinal carriage of ESBL producers in the community has been reported in Spain and Poland during the first years of the 21st century [28].

To the best of our knowledge, in Bulgaria, the present work is the first study on the faecal carriage of ESBL-producing enterobacteria in the community. It covered the period January 2018–March 2019 and revealed the intestinal carriage rates of ESBL and plasmidic AmpC producers in the community in 14.6% and 2.5% of the subjects, respectively. There was not any statistical significance in the differences between the adults and children. These data are worrisome, as the percentage of community ESBL carriers in Bulgaria (14.6%) was higher compared to those in many other European countries such as Hungary (3%) [29], England (11.3%) [30], Switzerland (5.8%) [31], and France (6%) [32]. In contrast, in Asia, the carriage was higher compared to our results. Data from Thailand showed a high frequency (61.7%) of patients with ESBL-producing isolates [33]. In another study from Thailand, CTX-M faecal carriage in the community was 65.7% [34]. Interestingly, in Japan, there was a much lower frequency (6.4%) of ESBL producers among healthy carriers, despite this country also being in Asia [35]. In China, large differences have been reported, ranging from 7% in Shenyang Province in 2007 to 50% in Fujian Province in 2009 [36,37]. The frequency of *bla*_ESBL_-positive isolates in our study was similar to that found in a large-scale study covering the entire world in 1978-2015, which revealed ESBL colonisation in 14% of healthy subjects and an increasing trend over time [38]. These findings show the need for strong monitoring of the frequency of resistant enterobacteria in the gut over time.

One of the reasons for the higher frequency of ESBL genes in Bulgaria compared with other countries could be the increased antibiotic usage, especially of cephalosporins. For the period 2018–2019, the European Centre for Disease Prevention and Control (ECDC) observed an increasing trend for overall antibiotic usage in Bulgaria with 20.7 defined daily dose (DDD) per 1000 inhabitants per day in 2019 vs. 17.2 in 2010. It was slightly above the average value (19.4 DDD per 1000 inhabitants per day) for Europe in 2019 [35]. The fact that the antibiotic usage in Bulgaria still increased is worrisome; in 2022, it was 25.7 DDD per 1000 inhabitants per day, which ranks our country at the third place after Romania and Greece. The increase in the antibiotic usage from 2019 to 2022 was 22.1% [39]. This fact shows the possibility of an increase in dissemination of resistant enterobacteria not only in hospitals but also in the community, considering the intestinal carriage as an important reservoir.

In general, the most important risk factors for carriage of resistant gut bacteria include international travels [40] and long hospital stay [41]. Moreover, the fact that the faecal colonisation can persist for months to years after the first detection is also very important [41,42]. It has been reported that following the primary detection of ESBL producers in the intestinal tract, it persisted for 4 months in 61% of the cases, for 7 months in 56% of the cases, for two years in 19% of the cases, and for three or more years in 15% of the cases [41].

Resistant gut bacteria can be carried over from the hospital to the family. In most studies, the rate of intestinal colonisation with ESBL- or carbapenemase-producing *Enterobacterales* in the community was lower than that among hospital isolates. For example, in a study in Portugal, the faecal colonisation in healthy carriers was 2% [43], and this percentage was significantly lower than that found in the hospitals (17% at admission and 24% in hospitalized patients) [44]. The percentage detected in the present study (14.6% for ESBL production and 2.5% for AmpC) was much lower than that of the hospital isolates found in two Bulgarian studies (30.2 and 32.3% of ESBL producers in 2015 and 2018–2019, respectively), [10,11]. In England, the opposite trend was observed with the colonisation rate being much higher in the community (11.3%) compared to the hospitals (9%) [45].

The predominant isolates in the present study were *E. coli* (77.9%, 109/140 isolates), followed by *K. pneumoniae* (7.9%, 11/140). *E. coli* was the predominant gut species in many studies in Europe and all over the world [6,32,33,43,46,47,48,49,50].

In the present study, the prevalence of ESBL- and AmpC-producing *E. coli* among the community carriers is 15.2% (109/717). This percentage is similar to the global level of ESBL-producing *E. coli* (16.5%) in faecal samples in a meta-analysis in 2021 [8].

These results imply a high risk of dissemination of difficult-to-treat isolates in the community and confirm that the gut is a significant reservoir of ESBL producers.

In addition, our isolates had high resistance to non-beta-lactam antibiotics (26–42% for aminoglycosides, 49–57% for fluoroquinolones, and 50% for trimethoprim/sulfamethoxazole) (Figure 2). No carbapenem-resistant isolates were detected in the current study, in contrast to studies on intestinal carriers in Bulgarian hospitals [10,11]. The situation is even worse due to the increased use of cephalosporins, mainly of the third generation, not only in hospitals, but also in the community, especially after the introduction of the tablet form of cefixime and cefpodoxime. This could be one of the reasons for the selection of resistant intestinal bacteria. Overall, the antibiotic resistance of the isolates of the community carriers in the current study is lower than that observed in the hospital settings during the same time interval, 2018–2019 [10,11].

The most prevalent gene in the present study is *bla*_CTX-M-15_ (37.1%), followed by *bla*_CTX-M-3_ (19.2%). The members of the CTX-M-9 groups *bla*_CTX-M-14_ (10%) and *bla*_CTX-M-27_ (4.3%) were also detected. *E. coli* isolates presented the whole spectrum of observed in current study genes encoding ESBLs. The results show that this species can have great influence on the dissemination of resistant determinants. Investigated *K. pneumoniae* isolates were associated with *bla*_CTX-M-3_ (Table 2). In Europe, the commonly observed gene was *bla*_CTX-M-15_ (in France, the UK, and other countries) with the exception of Spain where CTX-M-9 and -14 alleles were predominant [51]. In Africa, studies have been limited, but a few studies have reported CTX-M-9 or -14 enzymes. Similarly, in China, CTX-M-14 was the common enzyme. In general, the most frequently detected enzymes were CTX-M-14 and CTX-M-15 [52,53,54].

The *bla*_CTX-M-27_ was detected in faecal samples with increased frequency in the last years both in the community and in the hospitals. The presence of *bla*_CTX-M-27_ in carriers suggests that it can be detected in clinical isolates as well. In Portugal, CTX-M-27 was detected in 29% of the intestinal carriers in hospitals [41]. In a previous study on faecal carriage among hospitalised patients in Bulgaria, similar results have been reported, with the percentage of *bla*_CTX-M-27_ being 11% [11].

An interesting finding is that 18 isolates (12.9%) were positive for *bla*_DHA-1_ or *bla*_CMY-2_. So far, there have been only a few reports on plasmidic AmpC detection in gut enterobacteria [55].

The wide spectrum of clones (Table 3 and Table 4) associated with ESBL production in the current study supports the fact that not only can a clonal spread, but also a horizontal plasmid or transfer by different mobile elements, play a significant role in the spread of ESBL producers. The same findings have been reported by other authors [5].

An interesting finding in the current study was the detection of pAmpC genes mainly among *E.coli* isolates. The frequency among all 717 subjects was 2.5% without statistically significant difference between the adults and children. The proportion of the observed isolates was 11.4%, mainly *bla*_DHA-1_ carriers. These findings show the role of the gut as an important reservoir of pAmpC. In Bulgaria, DHA-1 was observed for the first time in 2019 in an *E. cloacae* complex isolate from blood [17]. Interestingly, DHA-1 producers were rarely observed; for instance, in Varna, among 159 ESBL-/carbapenemase-producing *K. pneumoniae*, only 8 were DHA-1 producers [56]. From the CMY group, *bla*_CMY-4_ was commonly detected in NDM-1-producing clinical *K. pneumoniae* in Bulgaria [11]. The frequency of pAmpC carriers in the current study was significant and indicates that the intestinal tract could be a hidden source of isolates harbouring plasmid AmpC enzymes. In the literature, there are not many reports on the detection of pAmpC producers. One report from Iran described an increased frequency of DHA and CIT groups [57], and one study from Cyprus reported pAmpC in 3% of isolates but without detection of the groups and numbers of enzyme genes [58].

As for the epidemiologic typing, six different ST types (ST353, ST34, ST280, ST11, ST14, and ST2449) were detected in *K. pneumoniae*. They did not belong to high-risk clones except for ST14 and ST11. ST11 was detected in one *K. pneumoniae* isolate (*n* = 1). It was a DHA-1 producer. This is widely distributed international MLST type which is associated with the carriage of wide-range carbapenemases, commonly NDM and KPC [6].

The ST353 type was detected in a study in China [59], and all isolates were carriers of KPC-2 carbapenemase. This is important and indicates the ability of this clone to acquire other beta-lactamases, including carbapenemases. This clone has also been reported as a carrier of *bla*_CTX-M-15_ genes [60]. In current study, the isolates were carriers of *bla*_CTX-M-3_.

In the literature, ST14 has been repeatedly reported as one of the most common ST types of NDM-positive *K. pneumoniae* strains [61,62,63,64]. The isolate in the current study was a *bla*_CTX-M-15_ carrier.

A total of 56 ERIC types were identified in *E. coli* isolates (*n* = 92). The most common type was the ST131 type, belonging to the most virulent group, B2 (16.5%, 18/109). The finding that all ST131 isolates belonged to the hypervirulent clone O25b:H4 (*PabB*-positive) is worrisome. This clone is distributed worldwide among human extraintestinal pathogenic *E. coli* (ExPEC) strains and is associated with a wide range of community-acquired and nosocomial infections (mainly bloodstream and urinary tract infections) [65,66]. This clone was responsible for the rapid increase in beta-lactam resistance in *E. coli*, mainly due to the production of CTX-M-15 enzymes [66]. The clinical importance of the O25b:H4 clade has been highlighted by many studies that showed its high virulence potential [66,67]. The isolates belonging to the ST131 clone were carriers of a wide range of different *bla*_CTX-M_ genes, mainly *bla*_CTX-M-15_, and only single isolates had *bla*_CTX-M-3_ (*n* = 3), *bla*_CTX-M-27_ (*n* = 2) and *bla*_CTX-M-14_ (*n* = 1). One of them carried a gene, encoding pAmpC (*bla*_CMY-2_). This is in concordance with the reported diversification of the ST131 clone into the C1 and C2 subclades. C2 is associated with fluoroquinolone resistance and production of CTX-M-15. The strains of the C1 subclade produce CTX-M-27 and CTX-M-14 and from a new subclade C1-M27 producing only CTX-M-27 [68]. A study in two hospitals in Spain and France revealed results similar to those in the present study: a high frequency of ST131 *E. coli* producing predominantly CTX-M-15, followed by CTX-M-14 and CTX-M-27 [69]. In recent years, representatives of ST131 [70], as well as those producing CTX-M-27, were increasingly detected. They belonged to the C1-M27 cluster and were first found in Japan [71,72] and then in other countries (Thailand, Australia, Canada, USA, France, Italy, Germany, Netherlands, and Spain) [73,74,75]. In addition to the higher morbidity of this clone, some authors observed a long-term gut carriage of ST131 strains [76].

The second most frequent clonal complex, CC10, was represented by 4 MLST types: ST4981 (in 8 isolates), ST4238 (3), ST10 (4), and ST34 (1) in a total of 17 isolates. The prevalent ESBLs was *bla*_CTX-M-15_, followed by *bla*_CTX-M-3_ and *bla*_CTX-M-9_. In a study in China, isolates from this clone were also shown to carry NDM-1 metallo-beta-lactamase [59], which poses risks for its future spread.

Sequence type ST38 has usually been associated with phylotype D [75], which is consistent with the present results: 11 ST38 isolates in the present study from phylogroup D. This ST type was common in clinically significant ESBL-producing *E. coli* isolates [75]. The most prevalent ESBL type in this clonal complex was *bla*_CTX-M-14_, followed by *bla*_CTX-M-27_. Three isolates of ST38 cluster carried plasmid AmpC gene *bla*_DHA-1_. The predominant type ST38 was demonstrated in a study of *E. coli* isolates from humans and animals in Germany [77].

ST394 belonging to phylogroup D was a carrier of the *bla*_CTX-M-15._ In a study in Pakistan, ST394 was predominant, harbouring many enteroaggregative genes characteristic for diarrhoeagenic *E. coli* and an allele for fimH30, previously associated with the successful distribution of ST131 [78].

ST73 (three isolates in the present study from phylogroup B2) is also an important ExPEC clone, and it harboured *bla*_CTX-M-15_. It was associated with a pandemic spread of ExPEC *E. coli* clonal group O6-B2-ST73. This clone has been found in both humans and birds [79,80]. It was one of the main ST types in a study in hospitals in Spain and France [69].

Only one of the isolates was MLST type ST1196, related to phylogroup B1, an established pAmpC producer, positive for *bla*_DHA-1_. This clone was also found in a study in Myanmar, but the authors found that the isolate produced metallo-beta-lactamase NDM-5 [81].

Antimicrobial resistance is a major threat to human and animal health and was associated with up to 4.5 million deaths worldwide in 2019 [82]. The colonisation of the intestinal tract by resistant enterobacteria is a risk factor for their spread in both hospitals and communities. Many regimens have been suggested in the literature to eradicate colonisation by ESBL or/and carbapenemase producers. The results are conflicting, and there is currently no evidence that they are effective. Perhaps probiotics or faecal microbiota transplantation could work with better success [83,84]. Another possible approach is the use of lytic bacteriophages. Successful decolonisation by bacteriophages has already been clinically observed [82].

## 5. Conclusions

The percentage of intestinal carriage of *bla*_ESB_^L^-positive isolates among the Bulgarian community was found to be 14.6%, which is slightly above the average frequency in the European countries. No carbapenem-resistant isolates were detected. The predominant isolates were *E. coli* (77.9%), followed by *K. pneumoniae* (7.9%). *bla*_CTX-M-15_ was the most frequent type (37.1%) among our isolates, followed by *bla*_CTX-M-3_ (19.2%), *bla*_CTX-M-14_ (10%), and *bla*_CTX-M-27_ (4.3%). Moreover, we observed 2.5% of the community subjects to have carried pAmpC, which indicates that the gut can be an important source of pAmpC genes. The predominant type was *bla*_DHA-1_. The detection of the highly virulent O25b:H4 clone (16.5%) among the faecal isolates is worrisome since this clone is associated with high morbidity and mortality. The prevalence of ESBL- and AmpC-producing enterobacteria in the community implies the risk of increasingly difficult treatment of the associated bacterial infections. Periodic monitoring of faecal carriage of ESBL, carbapenemase, and AmpC production is necessary.

## Figures and Tables

**Table 1:** The frequency of third-generation cephalosporin-resistant enterobacteria according to the medical centres and species.

Species/Medical Centre (MC)	*E. coli*	*K. pneumoniae*	*E. cloacae* Complex	*C. freundii* Complex	*M. morganii*	*H. alvei*
MC—Pleven	14	2	4	1	0	0
MC—Burgas	31	2	1	2	0	0
MC 1—Sofia	39	5	1	2	2	0
MC 2—Sofia	5	0	0	0	0	1
MC 3—Sofia	20	2	2	1	0	3
Total numbers (percent)	109 (77.9%)	11 (7.9%)	8 (5.7%)	6 (4.3%)	2 (1.4%)	4 (2.9%)

**Table 2:** The frequency of *bla*_ESBL_ and *bla*_AmpC_ genes detected in this study.

Bacterial Species Detected Genes	*E. coli* *n* = 109 (%)	*K. pneumoniae n* = 11 (%)	*E. cloacae* Complex *n* = 8 (%)	*C. freundii* Complex *n* = 6 (%)	*M. morganii* *n* = 2 (%)	*H. alvei* *n* = 4 (%)	Total Number *n* = 140 (%)
*bla*_CTX-M-15_	47 (43.1%)	3 (27.2%)	-	2 (33%)	-	-	52 (37.1%)
*bla*_CTX-M-1_	3 (2.8%)	-	-	-	-	-	3 (2.1%)
*bla*_CTX-M-3_	20 (18.3%)	7 (63.6%)	-	-	-	-	27 (19.3%)
*bla*_CTX-M-9_	1 (0.9%)	-	-	-	-	-	1 (0.2%)
*bla*_CTX-M-14_	13 (11.9%)	-	1 (12%)	-	-	-	14 (10%)
*bla*_CTX-M-27_	6 (5.5%)	-	-	-	-	-	6 (4.3%)
*bla*_SHV-12_	2 (1.8%)	-	-	-	-	-	2 (1.4%)
*bla*_DHA-1_	15 (13.8%)	1 (9.0%)	-	-	-	-	16 (11.4%)
*bla*_CMY-2_	2 (1.8%)	-	-	-	-	-	2 (1.4%)
AmpC producer	-	-	7 (88%)	4 (67%)	2 (100%)	4 (100%)	17 (12.1%)

**Table 3:** ST types among *K. pneumoniae* and the corresponding genes encoding ESBls or plasmidic AmpC.

Genes Encoded ESBls/Plasmidic AmpC	MLST Type Number	ERIC Type	Total Number
*bla*_CTX-M-3_	ST353_2_; ST2449_1_; ND_4_	b*_n_*_= 2_; unique *_n_*_= 5_	7
*bla*_CTX-M-15_	ST34_1_; ST14_1_	unique *n* = 2	2
*bla*_DHA-1_	ST11_1_	unique *n* = 1	1

Abbreviations: unique—unique profile, ND—no data.

**Table 4:** Associations between *E. coli* ST types and *bla*_ESBLs_ and *bla*_pAmpC_ among 109 *E. coli* isolates.

Genes Encoded ESBls/Plasmidic AmpC	MLST Type Number/CC	Total Number
*bla*_CTX-M-15_	ST131_11_; CC10(ST4981_5_; ST10_2_); ST394_4_; ST2797_2_; ST73_3_; ST1057_2_; ST9298_1_; ST1196_1_; ST405_1_; ND_15_	47
*bla*_CTX-M-3_	ST131_3_; CC10(ST4981_2_; ST4238_3_; ST10_2_; ST34_1_); ST155_1_; ST295_1_; ST1993_1_; ND_6_	20
*bla*_CTX-M-1_	ST2797_1_; CC155(ST56_1_); ND_1_	3
*bla*_CTX-M-14_	ST131_1_; CC10(ST38_6_); CC155(ST56_1_); ST2178_1_; ST2617_1_; ND_3_	13
*bla*_CTX-M-9_	CC10(ST4981_1_)	1
*bla*_CTX-M-27_	ST131_2_; ST38_2_; ND_2_	6
*bla*_SHV-12_	ST8578_1_; ST93_1_;	2
*bla*_DHA-1_	ST4238_1_; ST38_3_; ST69_3_; ST7670_2_; ST648_1_;	15
*bla*_CMY-2_	ST131_1_; ST9298_1_;	2

**Table 5:** ESBLs, phylotypes, and MLST types of *E. coli* isolates according to the ERIC types.

ERIC Type	MLST Type	CC	Phylogroup	ESBL/AmpC Plasmid Genes	Total Number
A	131	131	B2	*bla*_CTX-M-15_ (11), *bla*_CTX-M-3_ (3), *bla*_CTX-M-27_ (2), *bla*_CTX-M-14_ (1), *bla*_CMY-2_ (1)	18
X	4981	10	A	*bla*_CTX-M-15_ (5), *bla*_CTX-M-3_ (2), *bla*_CTX-M-9_ (1)	8
Y	4238	10	A	*bla*_CTX-M-3_ (3), *bla*_DHA-1_ (1)	4
J	10	10	A	*bla*_CTX-M-15_ (2), *bla*_CTX-M-3_ (2)	4
V	34	10	A	*bla*_CTX-M-3_ (1)	1
S	38	38	D	*bla*_CTX-M-14_ (6), *bla*_CTX-M-27_ (2), *bla*_DHA-1_ (3)	11
M	394	394	D	*bla*_CTX-M-15_ (4)	4
K	69	69	D	*bla*_DHA-1_ (3)	3
E	2797	-	D	*bla*_CTX-M-15_ (2), *bla*_CTX-M-1_ (1)	3
I	73	73	B2	*bla*_CTX-M-15_ (3)	3
P	1057	14	B2	*bla*_CTX-M-15_ (2)	2
O	7670	-	D	*bla*_DHA-1_ (2)	2
D	9298	-	B2	*bla*_CTX-M-15_ (1), *bla*_CMY-2_ (1)	2
F1	56	155	B1	*bla*_CTX-M-14_ (1), *bla*_CTX-M-1_ (1),	2
Unique	155	155	B1	bla_CTX-M-3_ (1)	10
	2178	-	B1	*bla*_CTX-M-14_ (1)	
	2617	59	B1	*bla*_CTX-M-14_ (1)	
	295	23	B1	*bla*_CTX-M-3_ (1)	
	648	648	B1	*bla*_DHA-1_ (1)	
	8578	12	B2	*bla*_SHV-12_ (1)	
	93	168	A	*bla*_SHV-12_ (1)	
	1196	-	B1	*bla*_CTX-M-15_ (1)	
	405	405	D	*bla*_CTX-M-15_ (1)	
	1993	-	B2	*bla*_CTX-M-3_ (1)	
Unique	ND	-	A(14), B1(3) B2(1), D(14)	*bla*_CTX-M-15_ (15), *bla*_CTX-M-3_ (6), *bla*_DHA-1_(5), *bla*_CTX-M-14_ (3), *bla*_CTX-M-27_ (2), *bla*_CTX-M-1_ (1)	32

Abbreviations: unique—unique profile, CC—clonal complex, ND—no data.

## Data Availability

The original contributions presented in the study are included in the article/Supplementary Material, further inquiries can be directed to the corresponding author.

## Supplementary Materials

The following supporting information can be downloaded at https://www.mdpi.com/article/10.3390/microorganisms12091777/s1, Table S1: Primers for group-specific PCRs (ESBL and AmpC detection); Table S2: Sequencing primers (amplification and sequencing); Table S3.1: SMLST K.pneumoniae: primers and annealing temperature; Table S3.2: MLST *E. coli* (Achtman): primers and annealing temperature.
