# Supplementary material for: Extended-Spectrum Beta-Lactamase- and Plasmidic AmpC-Producing Enterobacterales among the Faecal Samples in the Bulgarian Community
Source: Microorganisms. 2024 Aug 28;12(9):1777. doi: 10.3390/microorganisms12091777 (PMC11433957; doi:10.3390/microorganisms12091777)
Supplement: Supplementary file 1 [file microorganisms-12-01777-s001.zip › microorganisms-3167508-supplementary.pdf]

Table S1. Primers for group specific PCRs (ESBL and AmpC detection)

Primers		Annealing t°C	Amplicon size	Reference
SHV	SHV-A: actgaatgaggcgcttcc<br>SHV-B: tcccgcagataaatcacc	61°C	297 bp	16
CTX-M	CTX-M-V: cvatgtgcagyaccagtaa<br>CTX-M-R: argtsaccagaaymagcgg	61°C	585 bp	16
CMY	CMY F : aacacactgattgcgtctgac<br>CMY R : ctgggcctcatcgtcagtta	61°C	1,226 bp	17
DHA	DHA F : aac ttt cac agg tgt gct ggg t<br>DHA R: ccg tac gca tac tgg ctt tgc	63°C	405 bp	17
FOX	FOX F : aac atg ggg tat cag gga gat g<br>FOX R : caa agc gcg taa ccg gat tgg	63°C	190 bp	17
MOX	MOX F: gct gct caa gga gca cag gat<br>MOX R: cac att gac ata ggt gtg gtg c	64ºC	520 bp	17
ACC	ACCF: aac agc ctc agc agc cgg tta<br>ACCR: ttc gcc gca atc atc cct agc	64ºC	346 bp	17
ERIC-PCR	ERIC1R : atg taa gct cct ggg gat tca c<br>ERIC2: aag taa gtg act ggg gtg agc	26oC/5cycles<br>45oC/30cycles	variable	19

Table S2. Sequencing primers (amplification and sequencing)

ESBL	Primers	Annealing t°C	Amplicon size	Reference
SHV	SHV-H: aacgccgggttattct<br>SHV-E:ttagcgttgccagtgctc	58°C	930bp	14
CTX-M-1 group	CTX-M-1/P1c: tcgtctcttccagaataagg<br>CTX-M-1/P2c: aaggagaaccaggaaccacg	56°C	1100bp	14
CTX-M-9 group	F: agggaatactgatgtaac<br>R: agatacgtgatctgatcc	52°C	1000bp	11
CMY-2 group	CMY-2-K : ggtgcaaatcaaaca cac<br>CMY-2-M : actgcagcaacgacgggc	61°C	1400bp	11

DHA	DHA-1A: ctgatgaaaaaatcgttatc<br>DHA-1B: attccagtgcactcaaaaata	52°C	1116 bp	15

Table S3.1. MLST *K.pneumoniae – primers and annealing temperature*

Gen	Primers	Annealing t°C	Reference
rpoB	**F:** GGCGAAATGGCWGAGAACCA<br>**R:** GAGTCTTCGAAGTTGTAACC	59°C	**19**
gapA	**F:** TGAAATATGACTCCACTCACGG<br>**R:** CTTCAGAAGCGGCTTTGATGGCTT	64°C	**19**
mdh	**F:** CCCAACTCGCTTCAGGTTCAG<br>**R:** CCGTTTTTCCCCAGCAGCAG	64°C	19
pgi	**pgi2F:** CTGCTGGCGCTGATCGGCAT<br>**pgi2R:** TTATAGCGGTTAATCAGGCCGT	64°C	19
phoE	**F:** ACCTACCGCAACACCGACTTCTTCGG<br>**R:** TGATCAGAACTGGTAGGTGAT	59°C	19
infB	**F:** CTCGCTGCTGGACTATATTCG<br>**R:** CGCTTTCAGCTCAAGAACTTC	59°C	19
tonB	**F:** CTTTATACCTCGGTACATCAGGTT<br>**R:** ATTCGCCGGCTGRGCRGAGAG	64°C	19

Table S3.2. MLST *E. coli (Achtman) – primers and annealing temperature*

Gen	Primers	Annealing t°C	Reference
adk	**F:** TCATCATCTGCACTTTCCGC<br>**R:** CCAGATCAGCGCGAACTTCA	60°C	18
fumC	**F:** TCACAGGTCGCCAGCGCTTC<br>**R:** TCCCGGCAGATAAGCTGTGG	64°C	18
gyrB	**F:** TCGGCGACACGGATGACGGC<br>**R:** GTCCATGTAGGCGTTCAGGG	68°C	18
icd	**F:**ATGGAAAGTAAAGTAGTTGTTCCGGCA CA<br>**R:** GGACGCAGCAGGATCTGTT	60°C	18

mdh	**F:** AGCGCGTTCTGTTCAAATGC<br>**R:** CAGGTTCAGAACTCTCTCTGT	60ºC	18
purA	**F:** TCGGTAACGGTGTTGTGCTG<br>**R:** CATACGGTAAGCCACGCAGA	64ºC	18
